# Inactivity‐mediated molecular adaptations: Insights from a preclinical model of physical activity reduction

**DOI:** 10.14814/phy2.70140

**Published:** 2024-11-28

**Authors:** Alice Meyer, Nicole Kim, Melissa Nguyen, Monica Misch, Kevin Marmo, Jacob Dowd, Christian Will, Milica Janosevic, Erin J. Stephenson

**Affiliations:** ^1^ Department of Anatomy, College of Graduate Studies Midwestern University Downers Grove Illinois USA; ^2^ Chicago College of Osteopathic Medicine Midwestern University Downers Grove Illinois USA; ^3^ Department of Biomedical Science, College of Graduate Studies Midwestern University Downers Grove Illinois USA; ^4^ Physical Therapy Program, College of Health Sciences Midwestern University Downers Grove Illinois USA; ^5^ Physician Assistant Program, College of Health Sciences Midwestern University Downers Grove Illinois USA; ^6^ College of Dental Medicine Illinois Midwestern University Downers Grove Illinois USA

**Keywords:** physical activity reduction, physical inactivity, skeletal muscle metabolism, skeletal muscle transcriptome

## Abstract

Insufficient physical activity is associated with increased relative risk of cardiometabolic disease and is an independent risk factor for mortality. Experimentally reducing physical activity rapidly induces insulin resistance, impairs glucose handling, and drives metabolic inflexibility. These adaptations manifest during the early stages of physical inactivity, even when energy balance is maintained, suggesting that inactivity‐mediated metabolic reprogramming is an early event that precedes changes in body composition. To identify mechanisms that promote metabolic adaptations associated with physical inactivity, we developed a mouse model of physical activity reduction that permits the study of inactivity in animals prior to the onset of overt changes in body composition. Adult mice were randomized into three groups: an inactive control group (standard rodent housing), an active control group (treadmill running 5 d/week for 6‐weeks), and an activity reduction group (treadmill running for 4‐weeks, followed by 2‐weeks of inactivity). Transcriptional profiling of gastrocnemius muscle identified seven transcripts uniquely altered by physical activity reduction compared to the inactive and active control groups. Most identified transcripts had reported functions linked to bioenergetic adaptation. Future studies will provide deeper characterization of the function(s) of each the identified transcripts while also determining how inactivity affects transcriptional regulation in other tissues.

## 1 INTRODUCTION

Physical inactivity is one of the leading risk factors for mortality, contributing to >7% of all‐cause and cardiovascular deaths annually (Katzmarzyk et al., 2022). The global health burden of physical inactivity is driven by a relationship between physical inactivity and an increased relative risk of developing a suite of noncommunicable diseases such as type‐2 diabetes, coronary heart disease, stroke, dementia, and several types of cancer (Katzmarzyk et al., 2022; Strain et al., 2024). Despite the well‐established benefits of regular physical activity on overall health and well‐being, approximately one third of the adult population and 81% of adolescents globally do not meet the minimum level of physical activity required for health (i.e., 60 min/day of moderate intensity aerobic activities on at least 3 days/week for children and adolescents or 150 min/week of moderate intensity aerobic activity for adults) (Bankoski et al., 2011; Ekelund et al., 2019; Guthold et al., 2020; Henson et al., 2013; Knaeps et al., 2018; Wijndaele et al., 2014; World Health Organization, 2020). In most regions of the world the prevalence of physical inactivity is greatest in girls and women (Guthold et al., 2018, 2020; World Health Organization, 2020), while among adults who are classified as being physically inactive, older adults are disproportionately affected (Strain et al., 2024). Thus, there is a large global population that would benefit from increasing their physical activity levels. Unfortunately, an increasing number of individuals face barriers that prevent them from engaging in exercise for its health benefits (Pedersen et al., 2021), thus the prevalence of physical inactivity (Katzmarzyk et al., 2022; Lee et al., 2012) and inactivity‐related disease (Chew et al., 2023; DALYs & Collaborators, 2017; Lin et al., 2020) continues to increase.

The past two decades has seen a strong push toward characterizing the physiology of physical inactivity to identify how inactivity drives disease (Pinto et al., 2023; Reidy et al., 1985; Sarto et al., 2023). This necessary first step has identified a core suite of metabolic adaptations common across multiple models of physical activity reduction. Step reduction (Bowden Davies et al., 2018; Knudsen et al., 1985; Krogh‐Madsen et al., 1985; McGlory et al., 2018; Reidy et al., 2019; Thyfault & Krogh‐Madsen, 1985) and bedrest (Alibegovic et al., 2009; Bergouignan et al., 2006, 2009; Rudwill et al., 2018; Shur et al., 2022; Stephens et al., 2011; Trim et al., 2023) protocols in humans and wheel lock (Appriou et al., 2019; Company et al., 2013; Kump & Booth, 2005a, 2005b; Laye et al., 2007; Rector et al., 2008; Ruegsegger et al., 2017; Teich et al., 2016) and cage size reduction (Fushiki et al., 1991; Mahmassani et al., 2020; Roemers et al., 2019; Siripoksup et al., 2024) protocols in animals demonstrate that a reduction in physical activity rapidly induces insulin resistance, impairs glucose handling and drives metabolic inflexibility. These early metabolic adaptations manifest even when energy balance is maintained (Rudwill et al., 2018; Trim et al., 2023), suggesting inactivity‐mediated metabolic reprogramming precedes changes in body composition. However, despite a growing interest in understanding the physiology of physical inactivity, knowledge of the molecular mechanisms that facilitate metabolic adaptations caused by physical inactivity remains limited.

The ability to identify mechanisms responsible for inactivity physiology has been limited, in part, by how physical inactivity is modeled in preclinical settings. Most laboratory animals are housed in environments that restrict activity while concurrently providing free and continuous access to food. Such housing conditions are thought to render animals metabolically morbid making them unsuitable for use as physically active control animals when strategies that further reduce physical activity are employed (i.e., cage size reduction or hind limb casting/unloading) (Martin et al., 2010). In contrast, while wheel lock models do address the need for animals to be active prior to initiating activity reduction, voluntary wheel running poses its own limitations, such as not being able to easily control the duration and/or intensity of physical activity animals undertake during the active phase of the protocol.

Acknowledging the utility and limitations of existing animal models of physical activity reduction, we developed a treadmill‐based rodent model of physical activity reduction. The protocol involves an initial active phase where exercise intensity, duration, and time of day can be tightly controlled, followed by an inactive phase that reduces physical activity without shrinking home cage size or inhibiting hindlimb mobility. Alongside both inactive and active control animals, this manuscript describes this new model of physical activity reduction and reports the transcriptional changes uniquely caused by physical activity reduction in skeletal muscle. We hypothesized that physical activity reduction would cause a unique transcriptional response compared to both active and inactive control animals and that the differentially expressed transcripts associated with physical activity reduction would have functions linked to metabolism.

## 2 MATERIALS AND METHODS

### 2.1 Animals, housing, and diet

Female and male C57BL/6NJ mice (strain #005304) were purchased from The Jackson Laboratory at 9 weeks‐of age ± 3 days. Mice were studied during early adulthood to limit any potentially confounding interactions between physical activity levels and growth and sexual maturation in younger mice, while also limiting any effects of aging on physical activity and other metabolic parameters previously reported to be altered in middle aged or older adult mice (Quintanilha et al., 2021; Slemenda et al., 1994). Upon arrival at the Midwestern University Animal Facility, mice were housed in a temperature‐ and humidity‐controlled environment (22.2°C, 40% relative humidity) in 7.5′W × 11.5′L × 5′H cages (Ancare #N10) containing corn cob bedding and standardized enrichment (i.e., a plastic tunnel and an iso‐BLOX square). Animals were maintained on a 12:12 h light: dark photoperiod cycle and unless otherwise specified, mice had free access to food (Teklad Global #2918; 24% kCal from protein, 18% kCal from fat, 58% kCal from carbohydrate, 3.1 kCal/g) and autoclaved tap water at all times. Throughout the study period, mice were weighed twice weekly using a precision balance under dynamic weighing conditions (Mettler Toledo #MS1602TS). The Midwestern University Institutional Animal Care and Use Committee approved all animal procedures in advance of the study (protocol #3180).

### 2.2 Treadmill setup and acclimation procedure

All treadmill exercise was conducted on a 5‐Lane Touchscreen Convertible Treadmill (Panlab Harvard Apparatus #76–0896) set at a 15° incline. Mice were initially acclimated to the treadmill by placing them on the stationary belt for ~5 min, after which time the belt was started at a speed of 6 m/min. Belt speed was progressively increased in increments of 0.6 m/min every minute until mice were running at a speed of 10 m/min, which they maintained for 5 min before being returned to their home cages. This acclimation protocol was repeated up to three times (separate days for each occurrence).

### 2.3 Physical activity reduction protocol

**FIGURE 1:**
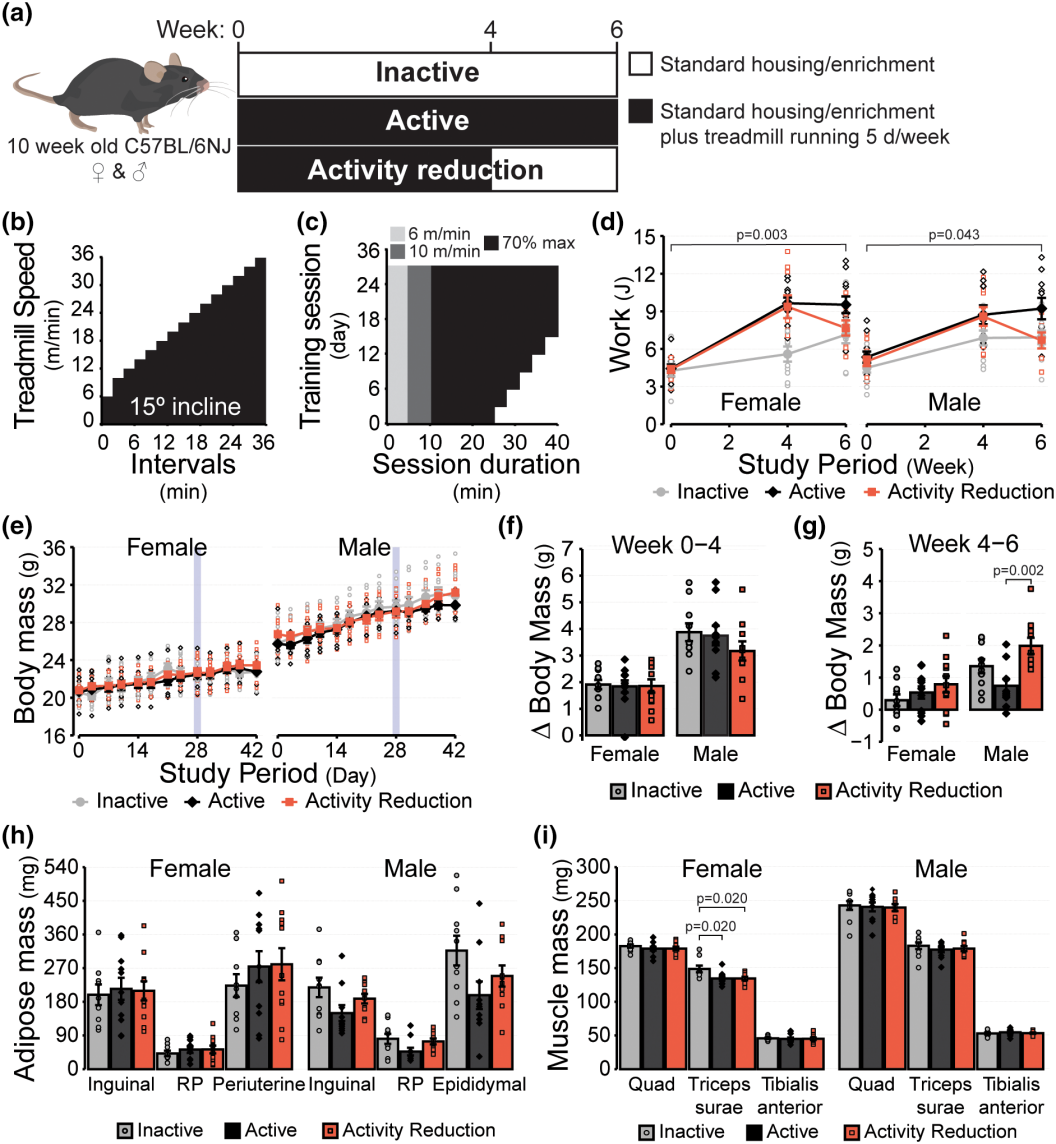
Activity reduction model. (a) Experimental design overview. (b) Overview of the graded maximal running test protocol, which was used to determine training speeds and verify performance adaptations at each stage of the study. (c) Overview of the treadmill training protocol. Training sessions occurred 5 d/week with two consecutive days of rest following every five consecutive days of training. (d) Work (J) calculated for individual mice after each of the graded maximal running tests. (e) Body mass of mice across the period of study. (f) Change in body mass between the start of the study and the end of the fourth week and (g) between the fourth week and the end of the study. (h) Mass of the inguinal, retroperitoneal (RP) and gonadal (periuterine and epididymal) adipose depots at the end of the study. (i) Mass of the quadriceps complex (Quad), triceps surae complex, and tibialis anterior skeletal muscle at the end of the study. Values reported are the mean ± standard error with individual values overlaid for *n* = 30 female (*n* = 10 Inactive, *n* = 11 Active, *n* = 9 Activity Reduction) and *n* = 30 male mice (*n* = 10 Inactive, *n* = 10 Active, *n* = 10 Activity Reduction). Data displayed in panels d and e were compared using linear mixed models with likelihood ratio tests. Data displayed in panels f‐h were compared using one‐way ANOVA with Tukey tests. Data in panel I was either compared by one‐way ANOVA (female quadriceps, male triceps surae, male tibialis anterior), or Kruskal‐Wallis tests with Dunn tests and Benjamini and Hochberg corrections (female triceps surae, female tibialis anterior, male quadriceps). Inactive group = light gray circles/bars, Active group = dark gray diamonds/bars, Activity Reduction group = red squares/bars.

Ten‐week‐old mice were randomized into one of three groups (Figure 1a). One group remained inactive (i.e., typical shoe‐box style housing with standard enrichment; inactive control group). A second group completed daily treadmill running sessions 5 d/week for 6 weeks (active control group), and a third group completed daily treadmill training 5 d/week for 4 weeks followed by 2 weeks of inactivity (activity reduction group). Each training session involved a 10‐min warm up period (5 min at 6 m/min, then 5 min at 10 m/min), followed by up to 30 min at ~70% of maximal running speed (predetermined during the prior graded maximal running test, described below; Figure 1b). Duration spent running at 70% of maximal running speed was progressively increased from an initial 15–30 min during the first 3 weeks of the training program and remained at 30 min/d thereafter (for a total of 40 min running per training session; Figure 1c). To promote training adaptations, running speed was increased by 5% after the first 2 weeks of training. After 4 weeks, graded maximal running tests were repeated and training speed was adjusted to 70% of the new maximal running speed. All training sessions were conducted between ZT 8–10 h. When necessary, mice were encouraged to run using vocal cues, bursts of compressed air, and/or use of a physical probe to gently tap their hindquarters.

### 2.4 Graded maximal running tests

Maximal running capacity was assessed at the beginning, after 4 weeks, and end of the study using graded maximal running tests. Mice were placed onto a stationary treadmill for up to 5 min after which the belt was started at a speed of 6 m/min. Mice maintained this speed for 2 min at which point the speed increased an additional 2 m/min every 2 min until mice reached exhaustion. Exhaustion was defined as the point when a mouse could no longer maintain a running pace that matched the speed of the treadmill and/or they refused to continue running despite gentle encouragement from the person(s) administering the test (as described above). Maximal speed, running distance, and running time were recorded. Work during each stage of the test was calculated and summed to determine the total work performed during each test. Work was calculated as: Work J = Force [Body mass kg × 9.18 N] × Vertical Distance [Speed m/sec × Incline (fractional grade) × Time sec].

### 2.5 Food intake

During the fifth week (i.e., after >1 week of inactivity for the activity reduction group), food intake of individual mice was determined. To facilitate measurement of food intake, mice were moved in pairs into 7.625 W” × 15.01” L × 5.13” H cages (Allentown #223581–4) with duel external bottle cage tops (Allentown #228843–1), stainless steel half pocket wire feeders (Allentown #314677) retrofitted with stainless steel dividers, and perforated plastic central dividers that physically separated mice and their food and water access points from one another while retaining many of the social aspects of group housing (Allentown #224520–1). Mice were provided with known amounts of food from which daily consumption was calculated based on the weight of food remaining on subsequent days. Food remaining included the chow pellets retained in the wire feeder basket and, where present, chow pellets and any obvious crumbs recovered from the cage bedding. Energy intake was calculated by multiplying the grams of food eaten by the energy density of the diet (3.1 kCal/g).

### 2.6 Bomb calorimetry

Stool was collected from cages following the final day of food intake monitoring and stored at −80°C. To prepare samples for calorimetry, stool was dried at 70°C in an oven overnight before being pressed into a single pellet of at least 25 mg using a pellet press. For each sample a 10 cm wire fuse was coiled, placed above the stool pellet and connected to the terminals of the calorimeter (6725 Semimicro Calorimeter, Parr). The bomb chamber was then filled with O_2_ to a pressure of 35 atm and stool pellets were combusted. Energy released during combustion was recorded.

### 2.7 Tissue collection

Mice were allowed to recover for 48–72 h following the final maximal running test after which they were fasted for 4–6 h starting at the beginning of the light photoperiod. Mice were anesthetized with isoflurane and blood was collected from the retroorbital sinus using a heparinized capillary and placed immediately on ice. Fasting blood glucose concentrations were measured using a hand‐held glucometer (Contour next ONE). Vaginal lavage with sterile PBS was performed on female mice to collect cells for microscopic identification and subsequent determination of estrous stage (McLean et al., 2012). Following blood collection, anesthetized mice were euthanized by cervical dislocation and tissues rapidly dissected. Right hindlimb muscles were immediately dissected out whole and freeze clamped between stainless steel paddles precooled in liquid nitrogen. Right hindlimb muscles (tibialis anterior and the quadriceps and triceps surae complexes) were carefully dissected out whole and weighed. The left inguinal, gonadal (periuterine or epididymal), and retroperitoneal adipose depots were carefully dissected out and weighed as indices of adiposity.

### 2.8 Insulin ELISA and HOMA‐IR

Fasting blood collected from the retroorbital sinus was allowed to rest on ice for at least 30 min before being centrifuged at 1000 RCF for 20 min at 4°C. Following separation of plasma from the cellular blood components, the plasma was moved into fresh tubes, snap frozen, and then stored at −80°C. Frozen plasma was thawed over ice and an ELISA was performed on samples and known standards in triplicate according to the manufacturer's instructions for a wide‐range assay (Crystal Chem #90080). Absorbance was read at 450 nm with background correction for absorbance at 630 nm (Spectramax iD5, Molecular Devices). Fasting plasma insulin concentrations and fasting blood glucose concentrations were used to calculate the Homeostasis Model Assessment of Insulin Resistance (HOMA‐IR) using the equation: HOMA‐IR = [fasting insulin (μU/mL) × fasting glucose (mmol/L)]/22.5.

### 2.9 Glycogen determination in skeletal muscle

The quadriceps muscle complex was powdered while frozen using a liquid nitrogen cooled mortar and pestle. Samples were placed in screw cap vials containing 30% KOH and boiled at 95°C for 30 min with occasional mixing. Samples were then cooled, and 1 M sodium sulfate and 100% ethanol were added. Samples were boiled again for 5 min before being centrifuged at 13,000 RCF for 5 min at room temperature. After discarding the supernatant, any residual glucose remaining on the glycogen pellet was washed away by resuspending the pellet in water, adding ethanol, and centrifuging the tubes to re‐pellet the glycogen. The wash step was repeated three times. Glycogen pellets were allowed to dry at room temperature before being digested overnight at 37°C in 0.3 mg/mL amyloglucosidase prepared in 50 mM sodium acetate, pH 4.8. The next morning, the amount of glucose derived from glycogen was quantified using a commercial glucose kit (Glucose Autokit, Wako diagnostics).

### 2.10 RNA isolation

RNA was extracted from the entire medial belly of the gastrocnemius muscles. The distribution of fiber types in the mouse gastrocnemius muscle (as determined by myosin‐ATPase activity) has previously been described as 5.7%–7.6% type‐I, 18.1%–22.7% type‐IIA, 21.7%–51.5% type‐IID/X, and 18.2%–54.4% type IIB fibers (Augusto et al., 2004; Manttari & Jarvilehto, 2005). Muscles underwent bead homogenization in Trizol. Chloroform was then added to each tube and homogenates were mixed and centrifuged at 4°C to separate the RNA from DNA and protein. RNA was collected from the upper phase and column purified using a commercial kit (PureLink RNA mini kit). RNA concentrations and quality were estimated spectrophotometrically (NanoDrop, ThermoFisher Scientific). RNA quality was determined by denaturing agarose gel electrophoresis (Aranda et al., 2012).

### 2.11 cDNA library prep and transcriptomics

Aliquots of RNA were sent for commercial sequencing (Azenta). Briefly, RNA integrity was verified by TapeStation (RIN range 8.8–9.8). RNA then underwent Poly(A) selection and cDNA libraries were prepared. Paired end bulk RNA seq was performed (Illumina). Data processing and analysis was completed in house as follows: FASTQ files were imported into Galaxy (The Galaxy Community, 2024) and sequencing data were trimmed (fastp [Chen et al., 2018]), aligned to genome assembly GRCm39 (RNA STAR [Dobin et al., 2012]), and reads mapped (featureCounts [Liao et al., 2013]). Count normalization and differential expression analysis was performed using DESeq2 (Love et al., 2014) in R. Transcripts were considered differentially expressed when FDR<0.05 and the fold change was >0.25 in either direction. Gene Set Enrichment Analysis (GSEA) was also performed (fgsea (Sergushichev, 2016)) using the Gene Ontology Biological Processes reference gene set which was downloaded from Molecular Signatures Database (Castanza et al., 2023; Liberzon et al., 2011; Subramanian et al., 2005). Enrichment of gene sets is reported where FDR <0.25.

### 2.12 Statistics

Time course data were analyzed by linear mixed models with likelihood ratio tests and (if applicable) inclusion of fixed factors in the models (see figure legends for details specific to each case), followed by pairwise comparisons using the estimated marginal means to identify differences between individual groups. Single observation data were assessed for homoskedasticity using Levene tests and for normality using Shapiro–Wilk tests. Data that met parametric assumptions were analyzed by either one‐way ANOVA with post hoc Tukey tests or ANCOVA. Nonparametric data were analyzed using Kruskal‐Wallis tests with post hoc Dunn tests. Data wrangling, statistical analyses, and data visualization were performed using R v4.4.1 in R Studio build 764 using the following packages: tidyverse v2.0.0 (Wickham et al., 2019), car v3.1–2 (Fox & Weisberg, 2019), rcompanion v2.4.36 (Mangiafico, 2024), rstatix v0.7.2 (Kassambara, 2023), lme4 v1.1–35.5 (Bates et al., 2015), and emmeans v1.10.4 (Lenth et al., 2024).

## 3 RESULTS

### 3.1 Running capacity

To ensure that the treadmill training protocol was sufficient to induce a training effect, graded maximal running tests were performed at 0‐, 4‐, and 6‐weeks and the amount of work performed during each test was determined (Figure 1d). Group effects were identified for both female (*p* = 0.003) and male mice (*p* = 0.043), with mice in the Active groups performing more overall work than mice in the Inactive groups (*p* = 0.003 for female and *p* = 0.044 for male mice). In contrast, work performed by the Activity Reduction groups was not different from that of the Active (*p* = 0.491 for female and *p* = 0.305 for male mice) or Inactive groups (*p* = 0.078 for female and *p* = 0.578 for male mice). These findings indicate that not only was the training protocol sufficient to improve performance in both female and male mice, but also that 2 weeks of inactivity following a four‐week training period was sufficient for reversing the effects of regular physical activity on performance.

### 3.2 Body mass

One goal in developing this model of activity reduction was to make sure data were collected after a period of reduced activity sufficient to ensure the effects being measured weren't simply the recovery response to the last exercise bout, but also not so long as to be confounded by significant body mass gains. To this end, a two‐week period of inactivity was chosen for the Activity Reduction groups and body mass was closely tracked throughout the study. Over the duration of the study, no differences in body mass were observed between groups for either sex (Figure 1e; *p* = 0.895 for female and *p* = 0.505 for male mice). Active male mice tended to weigh less than either the Inactive (−4.5%) or Activity Reduction (−4.3%) groups at the end of 6‐weeks, although this effect did not meet a priori assumptions for statistical significance (*p* = 0.220). The amount of body mass gained after the first 4 weeks also did not differ between groups for either sex (Figure 1f; *p* = 0.974 for female and *p* = 0.335 for male mice). However, between the fourth and sixth weeks, body mass gained by male mice was different between groups (Figure 1g, *p* = 0.003), with male mice in the Activity Reduction group having gained 167.5% more body mass than the Active group during this period (*p* = 0.002). The Inactive males also gained 82.1% more body mass than the Active males; however, this did not meet the a priori threshold for statistical significance (*p* = 0.165). Similarly, although the Activity Reduction males gained 46.9% more body mass than the Inactive males, this difference did not meet requirements for statistical significance either (*p* = 0.143). No differences were detected between groups for body mass gained between weeks four and six for female mice (*p* = 0.276). Together, these data indicate that although 2 weeks of activity reduction does not lead to a meaningful body mass divergence overall, activity reduction does rapidly reverse the effects of being physically active on suppressing body mass gains in male mice.

### 3.3 Adipose and skeletal muscle mass

Increases in adiposity and decreases in lean mass have been widely reported to occur in response to physical activity reduction (Company et al., 2013; Kump & Booth, 2005a; Laye et al., 2007; Roemers et al., 2019; Trim et al., 2023). Given these reports and the observation that male mice in the Activity Reduction group experienced catch up weight gain during the two‐week inactive phase of the study, we sought to determine whether the Activity Reduction protocol led to changes in the mass of a selection of adipose tissue depots and hind limb skeletal muscles (Figure 1h). For female mice, no differences were observed between groups for the mass of the inguinal, retroperitoneal, or periuterine adipose depots (*p* = 0.744, *p* = 0.481, and *p* = 0.365, respectively). For male mice, the Active group had less adipose mass in their inguinal, retroperitoneal, and epididymal depots when compared to males in the Inactive (−31.4%, −42.0%, and − 37.7%, respectively) and Activity Reduction groups (−20.4%, −36.6%, −20.6%, respectively). However, criteria for statistical significance were not quite met for any of the measured depots (*p* = 0.082, *p* = 0.077, and *p* = 0.074, respectively). Since both body mass and the mass of the individual adipose depots were highly variable within individual groups, this comparison was repeated with inclusion of body mass at the time of tissue collection as a covariate in a secondary analysis of the male mice. However, even with body mass included as a covariate, no statistically significant differences were detected between groups for either the inguinal, retroperitoneal, or epididymal depots (*p* = 0.112, *p* = 0.140, and *p* = 0.094, respectively). In both female and male mice, no differences between groups were observed for the mass of the quadriceps complex (*p* = 0.256 and *p* = 0.935, respectively) or the tibialis anterior muscle (*p* = 0.776 and *p* = 0.678). The triceps surae complex was also similar in mass across groups for male mice (*p* = 0.612); however, in female mice that were Active and those that underwent Activity Reduction, the triceps surae complex weighed less than that of the females in the Inactive group (12.9%, *p* = 0.020 and 12.9%, *p* = 0.020, respectively). Although limited by the adipose depots and hindlimb muscles chosen for comparison, and a lack of statistical power given the amount of variability within groups for the adipose depots, these data suggest that two‐weeks of Activity Reduction may alter body composition in a sexually dimorphic and tissue‐specific manner.

### 3.4 Energy intake and absorption

**FIGURE 2:**
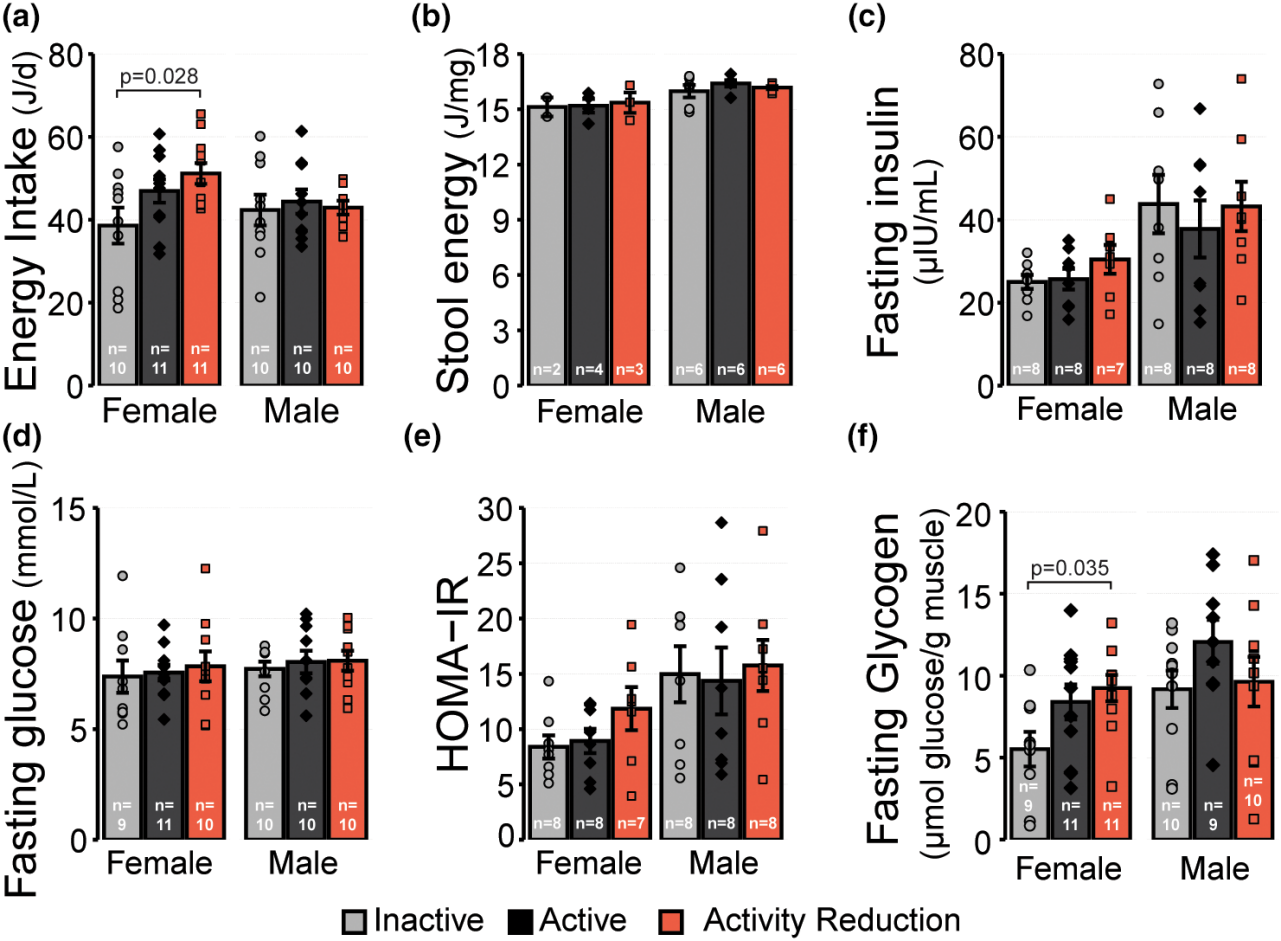
Effect of activity reduction on energy intake, glucose homeostasis and muscle glycogen stores. (a) Average daily energy intake from food in the final week of the study period. (b) Energy content of stool. (c) Fasting plasma insulin and (d) blood glucose concentrations, and (e) HOMA‐IR. (f) Glycogen content of the quadriceps muscles. Values reported are the mean ± standard error with individual values overlaid. Data displayed were compared using one‐way ANOVA with Tukey tests where appropriate. The number of biological replicates in each experiment is indicated on each individual panel. Inactive group = light gray circles/bars, Active group = dark gray diamonds/bars, Activity Reduction group = red squares/bars.

Following the observation that male mice experienced catch up body mass gains during the two‐week activity reduction period, we sought to determine whether any differences in body mass might be associated with changes in energy intake (Figure 2a) or energy absorption from the diet (Figure 2b). A group effect for energy intake was observed in female mice (*p* = 0.034), but not male mice (*p* = 0.876). Female mice in the Activity Reduction group consumed 32.6% more energy per day than females in the Inactive group (*p* = 0.028). Female mice in the Active group also had 21.5% greater daily energy intake than those in the Inactive group; however, this did not meet a priori criteria for statistical significance (*p* = 0.186). No differences in energy intake were observed between female mice in the Active and Activity Reduction groups (*p* = 0.618). The energy excreted in the stool was also measured as a surrogate measure of energy absorption from the diet (Figure 2b). No differences were observed between groups in either female (*p* = 0.941) or male mice (*p* = 0.438), suggesting that activity reduction had little effect on energy absorption. These findings suggest that in female but not male mice, being active likely regulates energy intake, an effect that persists into the first 2 weeks of activity reduction. Considering these observations alongside the earlier finding that activity reduction did not lead to differences in body mass or energy absorption in female mice, it's tempting to extrapolate these results to suggest that energy expenditure was likely greater in female mice in the Active and Activity Reduction groups compared to the Inactive group. In contrast, given that male mice in the Activity Reduction group were experiencing catch up weight gain during the period where energy intake and absorption were measured, and the observation that male mice across all three groups consumed and absorbed similar amounts of energy, we predict that male mice in the Activity Reduction group would have reduced energy expenditure compared to the Active and Inactive groups. Unfortunately, the experimental design used in this study does not allow for the accurate measurement of daily energy expenditure using indirect calorimetry. Thus, future studies using this model of activity reduction will need to consider alternative approaches to tease out the effects of activity reduction on energy expenditure and determine the if there are any sex‐specific responses.

### 3.5 Fasting insulin, glucose, and HOMA‐IR

**FIGURE 3:**
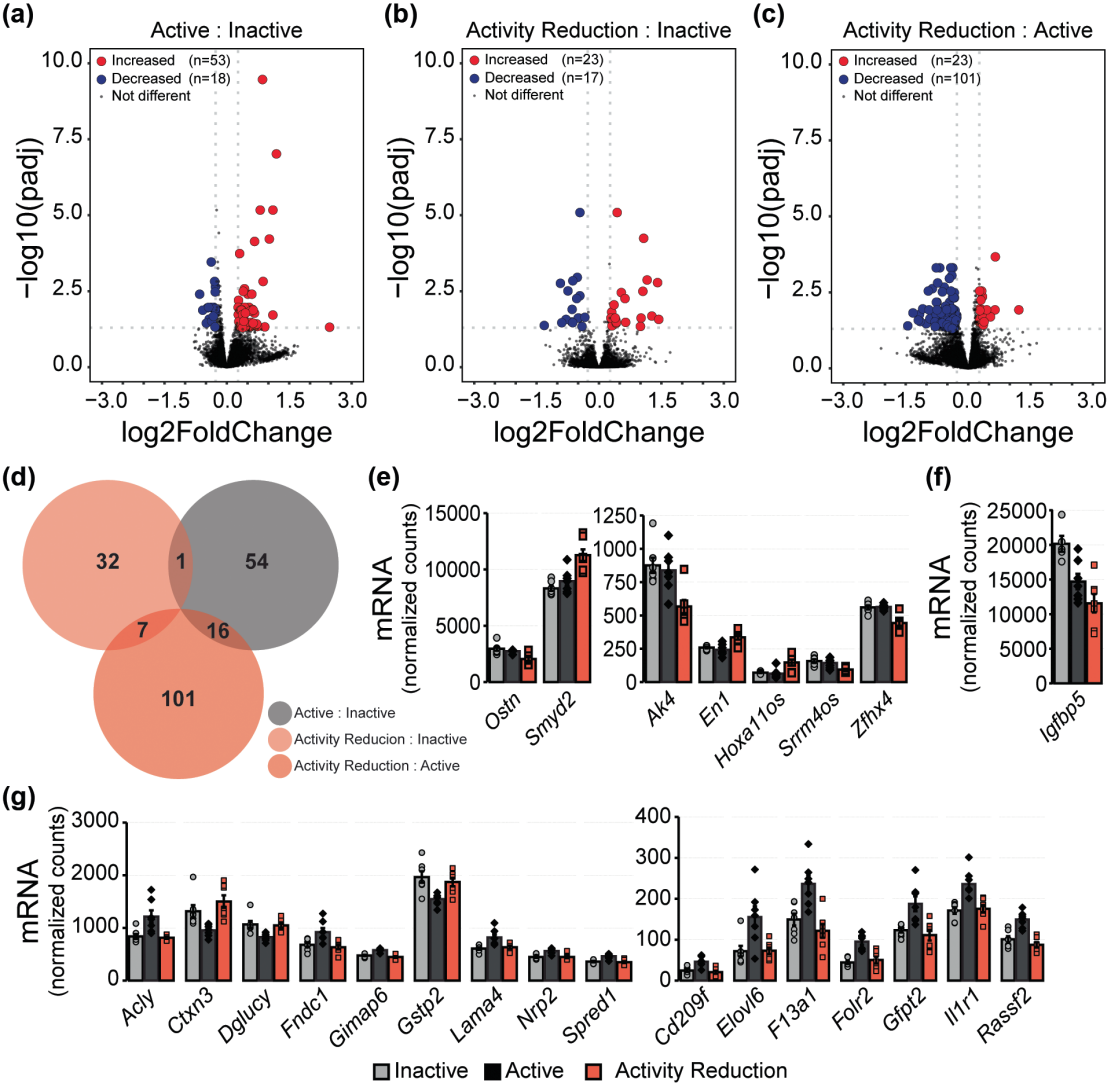
Effect of activity reduction on the skeletal muscle (gastrocnemius) transcriptome. Volcano plots demonstrate the number of up‐ and down‐regulated transcripts in (a) the Active compared to Inactive group, (b) the Activity Reduction compared to Inactive group, and (c) the Activity Reduction compared to Active group. Transcripts were considered differentially expressed when *q* < 0.05 and expression change was greater than or equal to 20% in either direction compared to the Inactive (a and b) or Active (c) groups. Blue = differentially expressed transcripts with reduced expression, red = differentially expressed transcripts with increased expression, gray = transcripts that are either not differentially expressed or are differentially expressed but do not meet the 20% threshold. (d) Venn diagram showing the overlapping and uniquely expressed transcripts in the Active versus Inactive group (gray), the Activity Reduction versus Inactive group (light red), and the Activity Reduction versus Active group (dark red). (e) Expression of transcripts uniquely changed by Activity Reduction compared to both the Inactive and Active groups. (f) *Igfbp5* expression is decreased in both the Active and Activity Reduction groups compared to the Inactive group. (g) Expression of transcripts uniquely changed by Activity compared to the Inactive and Activity Reduction groups. Values reported are the mean ± standard error with individual values overlaid for *n* = 21 female mice (*n* = 7 Inactive, *n* = 7 Active, *n* = 7 Activity Reduction). For panels e‐g, Inactive group = light gray circles/bars, Active group = dark gray diamonds/bars. Activity Reduction group = red squares/bars.

To determine whether two‐weeks of activity reduction was sufficient to induce hyperinsulinemia, hyperglycemia, insulin resistance, or other disruptions in glucose homeostasis that have been previously reported in other models of activity reduction (Alibegovic et al., 2009; Appriou et al., 2019; Bergouignan et al., 2009; Bowden Davies et al., 2018; Eggelbusch et al., 2024; Fushiki et al., 1991; Knudsen et al., 1985; Krogh‐Madsen et al., 1985; McGlory et al., 2018; Reidy et al., 2019; Rudwill et al., 2018; Shur et al., 2022; Siripoksup et al., 2024; Stephens et al., 2011; Trim et al., 2023), we measured fasting plasma insulin (Figure 3c), fasting blood glucose concentrations (Figure 3d), and calculated the HOMA‐IR index (Figure 3e). In both female and male mice, neither fasting insulin concentrations (*p* = 0.304 and *p* = 0.782, respectively) nor fasting glucose concentrations were different between groups (*p* = 0.860 and *p* = 0.808, respectively). HOMA‐IR was also not different in male mice (*p* = 0.932); however, there was a tendency for HOMA‐IR to be greater in female mice in the Activity Reduction group compared to the Active (32.8%) and Inactive groups (41.2%), although this did not meet the a priori threshold for statistical significance (*p* = 0.197). These findings suggest that 2 weeks of activity reduction was not sufficient to induce overt disruptions in systemic glucose homeostasis, at least not when animals are in a fasted state.

### 3.6 Muscle glycogen concentrations

Since skeletal muscle is a major organ of glucose uptake and storage in the form of glycogen, and a recent study indicated that bed rest in humans leads to increased muscle glycogen storage (Eggelbusch et al., 2024), we sought to determine whether activity reduction in mice also led to changes in the concentration of glycogen in the quadriceps muscle complex (Figure 3f). A group effect for muscle glycogen concentrations was observed for female mice (*p* = 0.037), which was explained by female mice in the Activity Reduction group having 67.6% more muscle glycogen than female mice in the Inactive group (*p* = 0.035). Muscle glycogen concentrations in the Active group were 52.7% greater than those of the Inactive group, but this difference did not meet a priori assumptions for statistical significance (*p* = 0.124). In contrast, although there was a tendency for male mice in the Active group to have greater muscle glycogen concentrations than those in the Inactive (31.5%) or Activity Reduction groups (25.2%), no group effect was detected (*p* = 0.292). These findings indicate that 2 weeks of physical activity reduction results in sexually dimorphic responses to muscle glycogen concentrations.

### 3.7 Transcriptional adaptations to activity reduction in skeletal muscle

A major goal of this study was to perform transcriptional profiling in tissues from mice that underwent activity reduction in the hopes of identifying transcripts uniquely changed in response to activity reduction compared to both active and chronically inactive control groups. Bulk RNAseq was performed on RNA isolated from the medial belly of the gastrocnemius muscles from female mice (Figure 3). The Active group had 71 differentially expressed transcripts compared to the Inactive group (Figure 3a), whereas the Activity Reduction group had 40 differentially expressed transcripts compared to the Inactive group (Figure 3b). Compared to the Active group, the Activity Reduction group had 124 differentially expressed transcripts (Figure 3c). After identifying the differentially expressed transcripts in each comparison, differentially expressed transcripts that either overlapped between or were unique to each comparison were determined (Figure 3d). Using this approach, seven transcripts were identified as being uniquely changed in the Activity Reduction group compared to both the Active and Inactive groups (Figure 3e). Of these transcripts, *Smyd2*, *En1*, and *Hoxa11os* all increased in expression following Activity Reduction, whereas *Ostn*, *Ak4*, *Srrm4os*, and *Zfhx4* all decreased in expression. One transcript, *Igfbp5*, was decreased in both the Active and Activity Reduction groups compared to the Inactive group (Figure 3f). Sixteen transcripts were differentially expressed in the Active group compared to both the Inactive and Activity Reduction group (Figure 3g), with the Active group having increased expression of *Acly*, *Cd209f*, *Elovl6*, *F13a1*, *Folr2*, *Fndc1*, *Gfpt2*, *Gimap6*, *Il1r1*, *Lama4*, *Nrp2*, *Rassf2*, and *Spred1* and decreased expression of *Ctxn3*, *Dglucy*, and *Gstp2*.

GSEA was also performed against the Gene Ontology Biological Processes curated gene set. Seventy‐six gene sets were positively enriched in the Active group compared to the Inactive group. Of these 76 gene sets, all were found to be negatively enriched in the Activity Reduction group when compared to the Active group, suggesting that enrichment of these pathways is dependent on regular physical activity and 2 weeks of physical activity reduction is sufficient to reverse the activity mediated adaptations. Pathways identified were involved in immune and stress responses, vasculature development and angiogenesis, wound repair, metabolism, and signal transduction. In addition to the pathways found to be positively enriched in response to activity and negatively enriched in response to activity reduction, seven other pathways were identified as being negatively enriched by activity reduction compared to the active group. These include *Endocytosis*, *Import into Cell*, *Vesicle mediated transport*, *Regulation of vesicle mediated transport*, *Regulation of catalytic activity*, *Positive regulation of catalytic activity*, and *Positive regulation of molecular function*. No pathway enrichment was observed when comparing the Activity Reduction group to the Inactive group.

## 4 DISCUSSION

Here, we report an animal model of physical activity reduction that acknowledges the need for animals to be active prior to initiating inactivity, but not so active that they only reflect the activity patterns of those at the higher end of the activity scale. This model offers an alternative to existing models of activity reduction, such as the cage size reduction (Fushiki et al., 1991; Mahmassani et al., 2020; Roemers et al., 2019; Siripoksup et al., 2024) and wheel lock models (Kump & Booth, 2005b; Tsai et al., 1981), where investigators are able to control the exercise intensity, exercise duration, and time of day that animals are active. This ensures the activity dose received by both the active control group and the activity reduction group (prior to initiation of inactivity) is consistent and at a volume that can be scaled up or down to reflect different training loads. Since traditional exercise training paradigms designed for increasing performance in humans typically involve exercising at 70% of maximal heart rate or 70% of maximal load, this study employed a training protocol that included a 10 min low speed warm up followed by 30 min running at 70% of maximal running speed 5 d per week. This approach resulted in performance adaptations following the initial four‐week training phase that then regressed to levels of the inactive control group following the two‐week period of activity reduction. Furthermore, by limiting the activity reduction phase to 2 weeks and including both active and inactive control groups in the experimental design, the molecular effects of physical activity reduction could be studied without the confounding influence of overt differences in body mass or body composition while ensuring effects specific to activity reduction could be distinguished from the mere reversal of any training‐specific adaptations. Limitations of this model also exist. Treadmill exercise is forced exercise which is arguably more stressful to mice compared to voluntary exercise such as wheel running. In the present study we aimed to mitigate the stress response by familiarizing animals to the treadmill ahead of any training sessions or testing of maximal running capacity. Training sessions were also held during the same 2‐h window at the end of each light cycle to minimize disruptions during the photoperiod mice are typically least active, and so mice could be familiarized to a routine. Additionally, tissues were collected 48–72 h after all mice had undergone the final maximal running test, thus mice in all three groups received the same treadmill stressor as well as a period of recovery to ensure the acute effects of treadmill exercise and any stress responses associated with it were controlled across all groups. However, whether any of the effects we observed were associated with more chronic adaptations to the stress of forced exercise cannot be ruled out.

Seven transcripts in skeletal muscle were identified as being uniquely changed in response to physical activity reduction. *Smyd2*, *En1*, and *Hoxa11os* were all increased in expression in response to activity reduction. Of these three transcripts, *Smyd2* was by far the most highly expressed. *Smyd2* encodes the lysine methyltransferase SET [Su(Var)3–9, Enhancer‐of‐zeste and Trithorax] and MYND [Myeloid, Nervy, and DEAF‐1] domain containing 2 (SMYD2). SMYD2 monomethylates lysine residues on both histone and nonhistone proteins (Rueda‐Robles et al., 2021) and has largely been studied in the context of tumor biology. Outside of cancer field, SMYD2 has been reported to play a role in regulating titin stability in striated muscle (Diehl et al., 2010; Donlin et al., 2012). More recently SMYD2 has been found to increase in abundance in the skeletal muscle and livers of hibernating animals during the early stages of torpor (Dang et al., 2023; Watts & Storey, 2019), providing further evidence that SMYD2 is induced when levels of physical activity are suddenly reduced. Given that SMYD2 has also been linked to the development of metabolic‐associated steatotic liver disease (Wang et al., 2023), we predict that SMYD2 plays a role in regulating metabolic adaptations caused by sudden reductions in bioenergetic demand. Indeed, based on findings in cervical cancer cell lines (Wang et al., 2021) and our own data (not shown, manuscript forthcoming), *Smyd2* induction in response to reductions in physical activity is likely linked to changes in the way glucose and glucose‐products are handled by skeletal muscle.

Although basal expression is much lower in skeletal muscle, *En1*, and *Hoxa11os* were also induced by activity reduction. *En1* encodes engrailed 1 (EN1), which has been shown to bind and regulate the expression of utrophin (Wang et al., 2011). *En1* has also been linked to pro‐fibrotic TGFβ signaling (Gyorfi et al., 2021; Mascharak et al., 2021), is highly expressed in dopaminergic neurons (Ma et al., 2024), and has been negatively associated with the thermogenic‐ and positively associated with the lipogenic capacities of brown adipocytes (Zhang et al., 2016). Less is known about the function of the long noncoding RNA *Hoxa11os*. However, in colon cells, *Hoxa11os* localizes to mitochondria and interacts with several core mitochondrial proteins involved in bioenergetic functions (Shmuel‐Galia et al., 2023). Thus, although the functions of *En1* and *Hoxa11os* in skeletal muscle are currently not well understood, these reports suggest *En1* and/or *Hoxa11os* induction in response to activity reduction could be linked to inactivity‐associated mitochondrial adaptations in one or more of the cell populations present in skeletal muscle.

Of the transcripts that were decreased in expression following activity reduction, *Ostn* had the highest basal expression. *Ostn* encodes a the musclin protein (also known as osteocrin), a known myokine that acts as an agonist for natriuretic peptide receptors (Nishizawa et al., 2004). *Ostn*/musclin induction in skeletal muscle is linked to energy availability, with *Ostn*/musclin expression found to be lower during fasting and higher upon refeeding (Nishizawa et al., 2004). *Ostn*/musclin is also induced acutely in response to treadmill running (Subbotina et al., 2015), indicating that it is exercise‐responsive and may contribute to adaptations associated with regular exercise. In line with this notion, when *Ostn*/musclin is absent, impairments in exercise capacity are observed, a finding thought to be due to musclin's purported influence over mitochondrial biogenesis (Subbotina et al., 2015). Conversely, musclin has been identified as a negative regulator of adipocyte thermogenesis (Jin et al., 2023), and high basal *Ostn*/musclin expression has been reported in animals and people with obesity, insulin resistance, and type‐2 diabetes (Chen, Liu, Sui, Yang, et al., 2017; Chen, Liu, Sui, Zhang, et al., 2017; Jin et al., 2023; Shimomura et al., 2021). In the present study, the expression of *Ostn* was reduced in response to physical activity reduction, as was overall exercise capacity compared to the Active control group. Although merely an association, given previous reports regarding the function of *Ostn*/musclin with regards to exercise tolerance (Subbotina et al., 2015), and its role in exercise‐mediated cardiac conditioning (Harris et al., 2023), it is possible that inhibition of *Ostn*/musclin is required for reversal of the effects of regular exercise on functions linked to exercise performance. Whether reductions in *Ostn* expression are associated with changes in circulating musclin concentrations, and whether low *Ostn* expression persists after longer periods of inactivity and/or once significant changes in body composition set in remains to be determined.

*Ak4*, *Srrm4os*, and *Zfhx4* were also all decreased in expression in response to activity reduction, although their basal expression was much lower than *Ostn*. *Ak4* encodes adenylate kinase 4 (AK4), a protein that localizes to the mitochondrial matrix (Noma et al., 2001) and is involved in regulating cellular AMP concentrations and, subsequently, the regulation of AMP‐sensitive metabolic enzymes such as the AMP‐activated Protein Kinase (i.e., AMPK) (Lanning et al., 2014). Thus, like *Ostn*/musclin, AK4 is also associated with adapting tissues to changes in energy availability. In the present study, physical activity reduction resulted in lower *Ak4* expression in skeletal muscle, an adaptation likely driven by the reduced bioenergetic flux that accompanies physical activity reduction.

Little is known about the function of *Zfhx4*, which encodes zinc finger homeobox 4. *Zfhx4* is highly expressed in developing muscle and proliferating myoblasts, but decreases in expression to very low levels in postnatal skeletal muscles and differentiated myotubes, suggesting its function(s) is/are not critical in tissues with large quantities of postmitotic cells, such as skeletal muscle (Kostich & Sanes, 1995). Indeed, ablation of *Zfhx4* leads to early postnatal lethality linked to respiratory failure, suggesting it plays an important role in early development (Zhang et al., 2021). In colorectal cancer cells, the expression of *Zfhx4* has been computationally associated with the expression of transcripts that promote fatty acid oxidation (Zou et al., 2024). Thus, considering the known effect of physical inactivity on lowering skeletal muscle fatty acid oxidation (Shur et al., 2022), and the observation that *Zfhx4* is reduced in expression following physical inactivity, it would not be completely out of line to consider a potential relationship between these processes. Future studies should investigate *Zfhx4* function in postnatal skeletal muscle and determine whether it plays a role in regulating fatty acid oxidation or other functions associated with adapting muscle to changes in bioenergetic demand.

*Srrm4os* encodes serine/arginine repetitive matrix 4, opposite strand. It exhibits low level expression across most tissues (Yue et al., 2014), although these screens did not include skeletal muscle which is where it was detected in this study and found it to be negatively regulated by physical activity reduction. The function of *Srrm4os* is yet to be determined.

## 5 CONCLUSION

Seven transcripts in skeletal muscle were identified as being uniquely altered by physical activity reduction. Although the functional contribution of each of the identified transcripts to the physiology of physical inactivity remains to be determined, this study was a necessary first step toward identifying potential mediators of the physiology associated with activity reduction and sets the stage for similar investigations in other tissues. By acknowledging the need for research animals to be more active than typical laboratory housing permits, but not so active the active animals only represent those at the higher end of the activity spectrum, while also ensuring that both active and inactive control groups are included in the experimental design, this work has opened the door to more nuanced investigations into the molecular mechanisms that drive the metabolic impairments associated with physical inactivity. Future studies will provide deeper characterization of the function(s) of each the seven transcripts identified as being uniquely altered by physical activity reduction in skeletal muscle. It is hoped that such next steps will result in identification of molecular targets that can be exploited for the development of therapeutics capable of supporting metabolism for the many people who cannot meet or sustain a level of physical activity required to protect against disease.

## AUTHOR CONTRIBUTIONS

EJS conceived and designed the research. ES, AM, MM, and KM trained the mice. ES, AM, MM, and MJ collected tissues from the mice. EJS, AM, NK, MN, MM, KM, JD, CW performed experiments. EJS, AM, and NK analyzed data. EJS, AM, and NK interpreted results of experiments. EJS prepared the figures. EJS and NK drafted the manuscript. EJS and AM edited and revised manuscript. All authors approved the submitted version of manuscript.

## FUNDING INFORMATION

This work was supported by a 2022 Midwestern University Faculty Seed Grant (to EJS) and 2022 and 2023 Midwestern University Core Outsourcing Awards (to EJS). NK was supported by a 2023 Kenneth A. Suarez Summer Research Fellowship.

## CONFLICT OF INTEREST STATEMENT

The authors currently have no disclosures, financial or otherwise.

## Data Availability

Sequencing files are available at Gene Expression Omnibus (accession number GSE281849). Source data and analysis code are available at https://github.com/esteph16/Inactivity‐mediated‐molecular‐adaptations‐1.

## References

[phy270140-bib-0001] Alibegovic, A. C. , Hojbjerre, L. , Sonne, M. P. , van Hall, G. , Stallknecht, B. , Dela, F. , & Vaag, A. (2009). Impact of 9 days of bed rest on hepatic and peripheral insulin action, insulin secretion, and whole‐body lipolysis in healthy young male offspring of patients with type 2 diabetes. Diabetes, 58, 2749–2756. 19720789 10.2337/db09-0369 PMC2780872

[phy270140-bib-0002] Appriou, Z. , Nay, K. , Pierre, N. , Saligaut, D. , Lefeuvre‐Orfila, L. , Martin, B. , Cavey, T. , Ropert, M. , Loreal, O. , Rannou‐Bekono, F. , & Derbre, F. (2019). Skeletal muscle ceramides do not contribute to physical‐inactivity‐induced insulin resistance. Applied Physiology, Nutrition, and Metabolism, 44, 1180–1188. 10.1139/apnm-2018-0850 30889368

[phy270140-bib-0003] Aranda, P. S. , LaJoie, D. M. , & Jorcyk, C. L. (2012). Bleach gel: A simple agarose gel for analyzing RNA quality. Electrophoresis, 33, 366–369. 22222980 10.1002/elps.201100335 PMC3699176

[phy270140-bib-0004] Augusto, V. , Padovani, C. R. , & Campos, G. E. R. (2004). Skeletal muscle fiber types in C57BL6J mice. Brazilian jounal of Morphology Sciences, 21, 89–94.

[phy270140-bib-0005] Bankoski, A. , Harris, T. B. , McClain, J. J. , Brychta, R. J. , Caserotti, P. , Chen, K. Y. , Berrigan, D. , Troiano, R. P. , & Koster, A. (2011). Sedentary activity associated with metabolic syndrome independent of physical activity. Diabetes Care, 34, 497–503. 21270206 10.2337/dc10-0987 PMC3024375

[phy270140-bib-0006] Bates, D. , Maechler, M. , Bolker, B. , & Walker, S. (2015). Fitting linear mixed‐effects models using lme4. Journal of Statistical Software, 67, 1–48.

[phy270140-bib-0007] Bergouignan, A. , Schoeller, D. A. , Normand, S. , Gauquelin‐Koch, G. , Laville, M. , Shriver, T. , Desage, M. , Le Maho, Y. , Ohshima, H. , Gharib, C. , & Blanc, S. (2006). Effect of physical inactivity on the oxidation of saturated and monounsaturated dietary fatty acids: Results of a randomized trial. PLoS Clinical Trials, 1, e27. 17016547 10.1371/journal.pctr.0010027 PMC1584255

[phy270140-bib-0008] Bergouignan, A. , Trudel, G. , Simon, C. , Chopard, A. , Schoeller, D. A. , Momken, I. , Votruba, S. B. , Desage, M. , Burdge, G. C. , Gauquelin‐Koch, G. , Normand, S. , & Blanc, S. (2009). Physical inactivity differentially alters dietary oleate and palmitate trafficking. Diabetes, 58, 367–376. 19017764 10.2337/db08-0263 PMC2628610

[phy270140-bib-0009] Bowden Davies, K. A. , Sprung, V. S. , Norman, J. A. , Thompson, A. , Mitchell, K. L. , Halford, J. C. G. , Harrold, J. A. , Wilding, J. P. H. , Kemp, G. J. , & Cuthbertson, D. J. (2018). Short‐term decreased physical activity with increased sedentary behaviour causes metabolic derangements and altered body composition: Effects in individuals with and without a first‐degree relative with type 2 diabetes. Diabetologia, 61, 1282–1294. 29671031 10.1007/s00125-018-4603-5

[phy270140-bib-0010] Castanza, A. S. , Recla, J. M. , Eby, D. , Thorvaldsdottir, H. , Bult, C. J. , & Mesirov, J. P. (2023). Extending support for mouse data in the molecular signatures database (MSigDB). Nature Methods, 20, 1619–1620. 37704782 10.1038/s41592-023-02014-7 PMC11397807

[phy270140-bib-0011] Chen, S. , Zhou, Y. , Chen, Y. , & Gu, J. (2018). Fastp: An ultra‐fast all‐in‐one FASTQ preprocessor. Bioinformatics, 34, i884–i890. 30423086 10.1093/bioinformatics/bty560 PMC6129281

[phy270140-bib-0012] Chen, W. J. , Liu, Y. , Sui, Y. B. , Yang, H. T. , Chang, J. R. , Tang, C. S. , Qi, Y. F. , Zhang, J. , & Yin, X. H. (2017). Positive association between musclin and insulin resistance in obesity: Evidence of a human study and an animal experiment. Nutrition & Metabolism (London), 14, 46. 10.1186/s12986-017-0199-x PMC5504815 28702069

[phy270140-bib-0013] Chen, W. J. , Liu, Y. , Sui, Y. B. , Zhang, B. , Zhang, X. H. , & Yin, X. H. (2017). Increased circulating levels of musclin in newly diagnosed type 2 diabetic patients. Diabetes & Vascular Disease Research, 14, 116–121. 28185530 10.1177/1479164116675493

[phy270140-bib-0014] Chew, N. W. S. , Ng, C. H. , Tan, D. J. H. , Kong, G. , Lin, C. , Chin, Y. H. , Lim, W. H. , Huang, D. Q. , Quek, J. , Fu, C. E. , Xiao, J. , Syn, N. , Foo, R. , Khoo, C. M. , Wang, J. W. , Dimitriadis, G. K. , Young, D. Y. , Siddiqui, M. S. , Lam, C. S. P. , … Muthiah, M. D. (2023). The global burden of metabolic disease: Data from 2000 to 2019. Cell Metabolism, 35, 414. 36889281 10.1016/j.cmet.2023.02.003

[phy270140-bib-0016] Company, J. M. , Roberts, M. D. , Toedebusch, R. G. , Cruthirds, C. L. , & Booth, F. W. (2013). Sudden decrease in physical activity evokes adipocyte hyperplasia in 70‐ to 77‐day‐old rats but not 49‐ to 56‐day‐old rats. American Journal of Physiology. Regulatory, Integrative and Comparative Physiology, 305, R1465–R1478. 24089381 10.1152/ajpregu.00139.2013 PMC3882693

[phy270140-bib-0017] DALYs, G. B. D. , & Collaborators, H. (2017). Global, regional, and national disability‐adjusted life‐years (DALYs) for 333 diseases and injuries and healthy life expectancy (HALE) for 195 countries and territories, 1990‐2016: A systematic analysis for the global burden of disease study 2016. Lancet, 390, 1260–1344. 28919118 10.1016/S0140-6736(17)32130-X PMC5605707

[phy270140-bib-0018] Dang, K. , Farooq, H. M. U. , Dong, J. , Yang, H. , Kong, Y. , Wang, H. , Jiang, S. , Gao, Y. , & Qian, A. (2023). Transcriptomic and proteomic time‐course analyses based on Metascape reveal mechanisms against muscle atrophy in hibernating Spermophilus dauricus. Comparative Biochemistry and Physiology. Part A, Molecular & Integrative Physiology, 275, 111336. 10.1016/j.cbpa.2022.111336 36280225

[phy270140-bib-0019] Diehl, F. , Brown, M. A. , van Amerongen, M. J. , Novoyatleva, T. , Wietelmann, A. , Harriss, J. , Ferrazzi, F. , Bottger, T. , Harvey, R. P. , Tucker, P. W. , & Engel, F. B. (2010). Cardiac deletion of Smyd2 is dispensable for mouse heart development. PLoS One, 5, e9748. 20305823 10.1371/journal.pone.0009748 PMC2840034

[phy270140-bib-0020] Dobin, A. , Davis, C. A. , Schlesinger, F. , Drenkow, J. , Zaleski, C. , Jha, S. , Batut, P. , Chaisson, M. , & Gingeras, T. R. (2012). STAR: ultrafast universal RNA‐seq aligner. Bioinformatics, 29, 15–21. 23104886 10.1093/bioinformatics/bts635 PMC3530905

[phy270140-bib-0021] Donlin, L. T. , Andresen, C. , Just, S. , Rudensky, E. , Pappas, C. T. , Kruger, M. , Jacobs, E. Y. , Unger, A. , Zieseniss, A. , Dobenecker, M. W. , Voelkel, T. , Chait, B. T. , Gregorio, C. C. , Rottbauer, W. , Tarakhovsky, A. , & Linke, W. A. (2012). Smyd2 controls cytoplasmic lysine methylation of Hsp90 and myofilament organization. Genes & Development, 26, 114–119. 22241783 10.1101/gad.177758.111 PMC3273835

[phy270140-bib-0022] Eggelbusch, M. , Charlton, B. T. , Bosutti, A. , Ganse, B. , Giakoumaki, I. , Grootemaat, A. E. , Hendrickse, P. W. , Jaspers, Y. , Kemp, S. , Kerkhoff, T. J. , Noort, W. , van Weeghel, M. , van der Wel, N. N. , Wesseling, J. R. , Frings‐Meuthen, P. , Rittweger, J. , Mulder, E. R. , Jaspers, R. T. , Degens, H. , & Wust, R. C. I. (2024). The impact of bed rest on human skeletal muscle metabolism. Cell Reports Medicine, 5, 101372. 38232697 10.1016/j.xcrm.2023.101372 PMC10829795

[phy270140-bib-0023] Ekelund, U. , Tarp, J. , Steene‐Johannessen, J. , Hansen, B. H. , Jefferis, B. , Fagerland, M. W. , Whincup, P. , Diaz, K. M. , Hooker, S. P. , Chernofsky, A. , Larson, M. G. , Spartano, N. , Vasan, R. S. , Dohrn, I. M. , Hagstromer, M. , Edwardson, C. , Yates, T. , Shiroma, E. , Anderssen, S. A. , & Lee, I. M. (2019). Dose‐response associations between accelerometry measured physical activity and sedentary time and all cause mortality: Systematic review and harmonised meta‐analysis. BMJ, 366, l4570. 31434697 10.1136/bmj.l4570 PMC6699591

[phy270140-bib-0024] Fox, J. , & Weisberg, S. (2019). An R companion to applied regression. Sage.

[phy270140-bib-0025] Fushiki, T. , Kano, T. , Inoue, K. , & Sugimoto, E. (1991). Decrease in muscle glucose transporter number in chronic physical inactivity in rats. The American Journal of Physiology, 260, E403–E410. 1672241 10.1152/ajpendo.1991.260.3.E403

[phy270140-bib-0026] Guthold, R. , Stevens, G. A. , Riley, L. M. , & Bull, F. C. (2018). Worldwide trends in insufficient physical activity from 2001 to 2016: A pooled analysis of 358 population‐based surveys with 1.9 million participants. The Lancet Global Health, 6, e1077–e1086. 30193830 10.1016/S2214-109X(18)30357-7

[phy270140-bib-0027] Guthold, R. , Stevens, G. A. , Riley, L. M. , & Bull, F. C. (2020). Global trends in insufficient physical activity among adolescents: A pooled analysis of 298 population‐based surveys with 1.6 million participants. Lancet Child Adolesc Health, 4, 23–35. 31761562 10.1016/S2352-4642(19)30323-2 PMC6919336

[phy270140-bib-0028] Gyorfi, A. H. , Matei, A. E. , Fuchs, M. , Liang, C. , Rigau, A. R. , Hong, X. , Zhu, H. , Luber, M. , Bergmann, C. , Dees, C. , Ludolph, I. , Horch, R. E. , Distler, O. , Wang, J. , Bengsch, B. , Schett, G. , Kunz, M. , & Distler, J. H. W. (2021). Engrailed 1 coordinates cytoskeletal reorganization to induce myofibroblast differentiation. The Journal of Experimental Medicine, 218, e20201916. 34259830 10.1084/jem.20201916 PMC8288503

[phy270140-bib-0029] Harris, M. P. , Zeng, S. , Zhu, Z. , Lira, V. A. , Yu, L. , Hodgson‐Zingman, D. M. , & Zingman, L. V. (2023). Myokine Musclin is critical for exercise‐induced cardiac conditioning. International Journal of Molecular Sciences, 24, 6525. 37047496 10.3390/ijms24076525 PMC10095193

[phy270140-bib-0030] Henson, J. , Yates, T. , Biddle, S. J. , Edwardson, C. L. , Khunti, K. , Wilmot, E. G. , Gray, L. J. , Gorely, T. , Nimmo, M. A. , & Davies, M. J. (2013). Associations of objectively measured sedentary behaviour and physical activity with markers of cardiometabolic health. Diabetologia, 56, 1012–1020. 23456209 10.1007/s00125-013-2845-9

[phy270140-bib-0031] Jin, L. , Han, S. , Lv, X. , Li, X. , Zhang, Z. , Kuang, H. , Chen, Z. , Lv, C. A. , Peng, W. , Yang, Z. , Yang, M. , Mi, L. , Liu, T. , Ma, S. , Qiu, X. , Wang, Q. , Pan, X. , Shan, P. , Feng, Y. , … Meng, Z. X. (2023). The muscle‐enriched myokine Musclin impairs beige fat thermogenesis and systemic energy homeostasis via Tfr1/PKA signaling in male mice. Nature Communications, 14, 4257. 10.1038/s41467-023-39710-z PMC10356794 37468484

[phy270140-bib-0032] Kassambara, A. (2023). rstatix: Pipe‐friendly framework for basic statistical tests.

[phy270140-bib-0033] Katzmarzyk, P. T. , Friedenreich, C. , Shiroma, E. J. , & Lee, I. M. (2022). Physical inactivity and noncommunicable disease burden in low‐income, middle‐income and high‐income countries. British Journal of Sports Medicine, 56, 101–106. 33782046 10.1136/bjsports-2020-103640 PMC8478970

[phy270140-bib-0034] Knaeps, S. , Bourgois, J. G. , Charlier, R. , Mertens, E. , Lefevre, J. , & Wijndaele, K. (2018). Ten‐year change in sedentary behaviour, moderate‐to‐vigorous physical activity, cardiorespiratory fitness and cardiometabolic risk: Independent associations and mediation analysis. British Journal of Sports Medicine, 52, 1063–1068. 27491779 10.1136/bjsports-2016-096083 PMC6089204

[phy270140-bib-0035] Knudsen, S. H. , Hansen, L. S. , Pedersen, M. , Dejgaard, T. , Hansen, J. , Hall, G. V. , Thomsen, C. , Solomon, T. P. , Pedersen, B. K. , & Krogh‐Madsen, R. (1985). Changes in insulin sensitivity precede changes in body composition during 14 days of step reduction combined with overfeeding in healthy young men. Journal of Applied Physiology, 113(7–15), 2012–2015. 10.1152/japplphysiol.00189.2011 22556394

[phy270140-bib-0036] Kostich, W. A. , & Sanes, J. R. (1995). Expression of zfh‐4, a new member of the zinc finger‐homeodomain family, in developing brain and muscle. Developmental Dynamics, 202, 145–152. 7537552 10.1002/aja.1002020206

[phy270140-bib-0037] Krogh‐Madsen, R. , Thyfault, J. P. , Broholm, C. , Mortensen, O. H. , Olsen, R. H. , Mounier, R. , Plomgaard, P. , van Hall, G. , Booth, F. W. , & Pedersen, B. K. (1985). A 2‐wk reduction of ambulatory activity attenuates peripheral insulin sensitivity. Journal of Applied Physiology, 108, 1034–1040. 10.1152/japplphysiol.00977.2009 20044474

[phy270140-bib-0038] Kump, D. S. , & Booth, F. W. (2005a). Sustained rise in triacylglycerol synthesis and increased epididymal fat mass when rats cease voluntary wheel running. The Journal of Physiology, 565, 911–925. 15774517 10.1113/jphysiol.2005.084525 PMC1464554

[phy270140-bib-0039] Kump, D. S. , & Booth, F. W. (2005b). Alterations in insulin receptor signalling in the rat epitrochlearis muscle upon cessation of voluntary exercise. The Journal of Physiology, 562, 829–838. 15550465 10.1113/jphysiol.2004.073593 PMC1665545

[phy270140-bib-0040] Lanning, N. J. , Looyenga, B. D. , Kauffman, A. L. , Niemi, N. M. , Sudderth, J. , DeBerardinis, R. J. , & MacKeigan, J. P. (2014). A mitochondrial RNAi screen defines cellular bioenergetic determinants and identifies an adenylate kinase as a key regulator of ATP levels. Cell Reports, 7, 907–917. 24767988 10.1016/j.celrep.2014.03.065 PMC4046887

[phy270140-bib-0041] Laye, M. J. , Thyfault, J. P. , Stump, C. S. , & Booth, F. W. (2007). Inactivity induces increases in abdominal fat. Journal of Applied Physiology, 102, 1341–1347. 17122374 10.1152/japplphysiol.01018.2006

[phy270140-bib-0042] Lee, I. M. , Shiroma, E. J. , Lobelo, F. , Puska, P. , Blair, S. N. , Katzmarzyk, P. T. , & Lancet Physical Activity Series Working G . (2012). Effect of physical inactivity on major noncommunicable diseases worldwide: An analysis of burden of disease and life expectancy. Lancet, 380, 219–229. 22818936 10.1016/S0140-6736(12)61031-9 PMC3645500

[phy270140-bib-0043] Lenth, R. V. , Bolker, B. , Buerkner, P. , Giné‐Vázquez, I. , Herve, M. , Jung, M. , Love, J. , Miguez, F. , Piaskowski, J. , Riebl, H. , & Singmann, H. (2024). emmeans: Estimated Marginal Means, aka Least‐Squares Means.

[phy270140-bib-0044] Liao, Y. , Smyth, G. K. , & Shi, W. (2013). featureCounts: An efficient general purpose program for assigning sequence reads to genomic features. Bioinformatics, 30, 923–930. 24227677 10.1093/bioinformatics/btt656

[phy270140-bib-0045] Liberzon, A. , Subramanian, A. , Pinchback, R. , Thorvaldsdottir, H. , Tamayo, P. , & Mesirov, J. P. (2011). Molecular signatures database (MSigDB) 3.0. Bioinformatics, 27, 1739–1740. 21546393 10.1093/bioinformatics/btr260 PMC3106198

[phy270140-bib-0046] Lin, X. , Xu, Y. , Pan, X. , Xu, J. , Ding, Y. , Sun, X. , Song, X. , Ren, Y. , & Shan, P. F. (2020). Global, regional, and national burden and trend of diabetes in 195 countries and territories: An analysis from 1990 to 2025. Scientific Reports, 10, 14790. 32901098 10.1038/s41598-020-71908-9 PMC7478957

[phy270140-bib-0093] Love, M. I. , Huber, W. , & Anders, S. (2014). Moderated estimation of fold change and dispersion for RNA‐seq data with DESeq2. Genome Biology, 15, 550. 25516281 10.1186/s13059-014-0550-8 PMC4302049

[phy270140-bib-0047] Ma, X. , Zhao, L. L. , Yu, Y. C. , & Cheng, Y. (2024). Engrailed: Pathological and physiological effects of a multifunctional developmental gene. Genesis, 62, e23557. 37830136 10.1002/dvg.23557

[phy270140-bib-0048] Mahmassani, Z. S. , Reidy, P. T. , McKenzie, A. I. , Petrocelli, J. J. , Matthews, O. , de Hart, N. M. , Ferrara, P. J. , O'Connell, R. M. , Funai, K. , & Drummond, M. J. (2020). Absence of MyD88 from skeletal muscle protects female mice from inactivity‐induced adiposity and insulin resistance. Obesity (Silver Spring), 28, 772–782. 32108446 10.1002/oby.22759 PMC7093260

[phy270140-bib-0049] Mangiafico, S. (2024). Rcompanion: Functions to support extension education program evaluation. The Comprehensive R Archive Network. https://cran.r‐project.org/web/packages/rcompanion/

[phy270140-bib-0050] Manttari, S. , & Jarvilehto, M. (2005). Comparative analysis of mouse skeletal muscle fibre type composition and contractile responses to calcium channel blocker. BMC Physiology, 5, 4. 15710036 10.1186/1472-6793-5-4 PMC550649

[phy270140-bib-0051] Martin, B. , Ji, S. , Maudsley, S. , & Mattson, M. P. (2010). “control” laboratory rodents are metabolically morbid: Why it matters. Proceedings of the National Academy of Sciences of the United States of America, 107, 6127–6133. 20194732 10.1073/pnas.0912955107 PMC2852022

[phy270140-bib-0052] Mascharak, S. , desJardins‐Park, H. E. , Davitt, M. F. , Griffin, M. , Borrelli, M. R. , Moore, A. L. , Chen, K. , Duoto, B. , Chinta, M. , Foster, D. S. , Shen, A. H. , Januszyk, M. , Kwon, S. H. , Wernig, G. , Wan, D. C. , Lorenz, H. P. , Gurtner, G. C. , & Longaker, M. T. (2021). Preventing Engrailed‐1 activation in fibroblasts yields wound regeneration without scarring. Science, 372, eaba2374. 33888614 10.1126/science.aba2374 PMC9008875

[phy270140-bib-0053] McGlory, C. , von Allmen, M. T. , Stokes, T. , Morton, R. W. , Hector, A. J. , Lago, B. A. , Raphenya, A. R. , Smith, B. K. , McArthur, A. G. , Steinberg, G. R. , Baker, S. K. , & Phillips, S. M. (2018). Failed recovery of glycemic control and Myofibrillar protein synthesis with 2 wk of physical inactivity in overweight, Prediabetic older adults. The Journals of Gerontology. Series A, Biological Sciences and Medical Sciences, 73, 1070–1077. 29095970 10.1093/gerona/glx203 PMC6037059

[phy270140-bib-0054] McLean, A. C. , Valenzuela, N. , Fai, S. , & Bennett, S. A. (2012). Performing vaginal lavage, crystal violet staining, and vaginal cytological evaluation for mouse estrous cycle staging identification. Journal of Visualized Experiments, e4389. 10.3791/4389 PMC3490233 23007862

[phy270140-bib-0055] Nishizawa, H. , Matsuda, M. , Yamada, Y. , Kawai, K. , Suzuki, E. , Makishima, M. , Kitamura, T. , & Shimomura, I. (2004). Musclin, a novel skeletal muscle‐derived secretory factor. The Journal of Biological Chemistry, 279, 19391–19395. 15044443 10.1074/jbc.C400066200

[phy270140-bib-0056] Noma, T. , Fujisawa, K. , Yamashiro, Y. , Shinohara, M. , Nakazawa, A. , Gondo, T. , Ishihara, T. , & Yoshinobu, K. (2001). Structure and expression of human mitochondrial adenylate kinase targeted to the mitochondrial matrix. The Biochemical Journal, 358, 225–232. 11485571 10.1042/0264-6021:3580225 PMC1222051

[phy270140-bib-0057] Pedersen, M. R. L. , Hansen, A. F. , & Elmose‐Osterlund, K. (2021). Motives and barriers related to physical activity and sport across social backgrounds: Implications for health promotion. International Journal of Environmental Research and Public Health, 18, 5810. 34071630 10.3390/ijerph18115810 PMC8198157

[phy270140-bib-0058] Pinto, A. J. , Bergouignan, A. , Dempsey, P. C. , Roschel, H. , Owen, N. , Gualano, B. , & Dunstan, D. W. (2023). Physiology of sedentary behavior. Physiological Reviews, 103, 2561–2622. 37326297 10.1152/physrev.00022.2022 PMC10625842

[phy270140-bib-0059] Quintanilha, A. C. S. , Benfato, I. D. , Santos, R. L. O. , Antunes, H. K. M. , & de Oliveira, C. A. M. (2021). Effects of acute exercise on spontaneous physical activity in mice at different ages. BMC Sports Science, Medicine and Rehabilitation, 13, 78. 10.1186/s13102-021-00311-2 PMC8317422 34315514

[phy270140-bib-0060] Rector, R. S. , Thyfault, J. P. , Laye, M. J. , Morris, R. T. , Borengasser, S. J. , Uptergrove, G. M. , Chakravarthy, M. V. , Booth, F. W. , & Ibdah, J. A. (2008). Cessation of daily exercise dramatically alters precursors of hepatic steatosis in Otsuka long‐Evans Tokushima fatty (OLETF) rats. The Journal of Physiology, 586, 4241–4249. 18617560 10.1113/jphysiol.2008.156745 PMC2652173

[phy270140-bib-0061] Reidy, P. T. , Monnig, J. M. , Pickering, C. E. , Funai, K. , & Drummond, M. J. (1985). Preclinical rodent models of physical inactivity‐induced muscle insulin resistance: Challenges and solutions. Journal of Applied Physiology, 130(537–544), 2021. 10.1152/japplphysiol.00954.2020 PMC7988796 33356986

[phy270140-bib-0062] Reidy, P. T. , Yonemura, N. M. , Madsen, J. H. , McKenzie, A. I. , Mahmassani, Z. S. , Rondina, M. T. , Lin, Y. K. , Kaput, K. , & Drummond, M. J. (2019). An accumulation of muscle macrophages is accompanied by altered insulin sensitivity after reduced activity and recovery. Acta Physiologica (Oxford, England), 226, e13251. 30632274 10.1111/apha.13251 PMC9250031

[phy270140-bib-0063] Roemers, P. , Hulst, Y. , van Heijningen, S. , van Dijk, G. , van Heuvelen, M. J. G. , De Deyn, P. P. , & van der Zee, E. A. (2019). Inducing physical inactivity in mice: Preventing climbing and reducing cage size negatively affect physical fitness and body composition. Frontiers in Behavioral Neuroscience, 13, 221. 31680890 10.3389/fnbeh.2019.00221 PMC6797814

[phy270140-bib-0064] Rudwill, F. , O'Gorman, D. , Lefai, E. , Chery, I. , Zahariev, A. , Normand, S. , Pagano, A. F. , Chopard, A. , Damiot, A. , Laurens, C. , Hodson, L. , Canet‐Soulas, E. , Heer, M. , Meuthen, P. F. , Buehlmeier, J. , Baecker, N. , Meiller, L. , Gauquelin‐Koch, G. , Blanc, S. , … Bergouignan, A. (2018). Metabolic inflexibility is an early marker of bed‐rest‐induced glucose intolerance even when fat mass is stable. The Journal of Clinical Endocrinology and Metabolism, 103, 1910–1920. 29546280 10.1210/jc.2017-02267 PMC7263792

[phy270140-bib-0065] Rueda‐Robles, A. , Audano, M. , Alvarez‐Mercado, A. I. , & Rubio‐Tomas, T. (2021). Functions of SMYD proteins in biological processes: What do we know? An updated review. Archives of Biochemistry and Biophysics, 712, 109040. 34555372 10.1016/j.abb.2021.109040

[phy270140-bib-0066] Ruegsegger, G. N. , Sevage, J. A. , Childs, T. E. , Grigsby, K. B. , & Booth, F. W. (2017). 5‐Aminoimidazole‐4‐carboxamide ribonucleotide prevents fat gain following the cessation of voluntary physical activity. Experimental Physiology, 102, 1474–1485. 28786140 10.1113/EP086335

[phy270140-bib-0067] Sarto, F. , Bottinelli, R. , Franchi, M. V. , Porcelli, S. , Simunic, B. , Pisot, R. , & Narici, M. V. (2023). Pathophysiological mechanisms of reduced physical activity: Insights from the human step reduction model and animal analogues. Acta Physiologica (Oxford, England), 238, e13986. 37178372 10.1111/apha.13986

[phy270140-bib-0068] Sergushichev, A. A. (2016). An algorithm for fast preranked gene set enrichment analysis using cumulative statistic calculation. bioRxiv. 10.1101/060012

[phy270140-bib-0069] Shimomura, M. , Horii, N. , Fujie, S. , Inoue, K. , Hasegawa, N. , Iemitsu, K. , Uchida, M. , & Iemitsu, M. (2021). Decreased muscle‐derived musclin by chronic resistance exercise is associated with improved insulin resistance in rats with type 2 diabetes. Physiological Reports, 9, e14823. 33955191 10.14814/phy2.14823 PMC8100388

[phy270140-bib-0070] Shmuel‐Galia, L. , Humphries, F. , Vierbuchen, T. , Jiang, Z. , Santos, N. , Johnson, J. , Shklyar, B. , Joannas, L. , Mustone, N. , Sherman, S. , Ward, D. , Houghton, J. , Baer, C. E. , O'Hara, A. , Henao‐Mejia, J. , Hoebe, K. , & Fitzgerald, K. A. (2023). The lncRNA HOXA11os regulates mitochondrial function in myeloid cells to maintain intestinal homeostasis. Cell Metabolism, 35, 1441. 37494932 10.1016/j.cmet.2023.06.019

[phy270140-bib-0071] Shur, N. F. , Simpson, E. J. , Crossland, H. , Chivaka, P. K. , Constantin, D. , Cordon, S. M. , Constantin‐Teodosiu, D. , Stephens, F. B. , Lobo, D. N. , Szewczyk, N. , Narici, M. , Prats, C. , Macdonald, I. A. , & Greenhaff, P. L. (2022). Human adaptation to immobilization: Novel insights of impacts on glucose disposal and fuel utilization. Journal of Cachexia, Sarcopenia and Muscle, 13, 2999–3013. 36058634 10.1002/jcsm.13075 PMC9745545

[phy270140-bib-0072] Siripoksup, P. , Cao, G. , Cluntun, A. A. , Maschek, J. A. , Pearce, Q. , Lang, M. J. , Jeong, M. Y. , Eshima, H. , Ferrara, P. J. , Opurum, P. C. , Mahmassani, Z. S. , Peterlin, A. D. , Watanabe, S. , Walsh, M. A. , Taylor, E. B. , Cox, J. E. , Drummond, M. J. , Rutter, J. , & Funai, K. (2024). Sedentary behavior in mice induces metabolic inflexibility by suppressing skeletal muscle pyruvate metabolism. The Journal of Clinical Investigation, 134, e167371. 38652544 10.1172/JCI167371 PMC11142742

[phy270140-bib-0073] Slemenda, C. W. , Reister, T. K. , Hui, S. L. , Miller, J. Z. , Christian, J. C. , & Johnston, C. C., Jr. (1994). Influences on skeletal mineralization in children and adolescents: Evidence for varying effects of sexual maturation and physical activity. The Journal of Pediatrics, 125, 201–207. 8040762 10.1016/s0022-3476(94)70193-8

[phy270140-bib-0074] Stephens, B. R. , Granados, K. , Zderic, T. W. , Hamilton, M. T. , & Braun, B. (2011). Effects of 1 day of inactivity on insulin action in healthy men and women: Interaction with energy intake. Metabolism, 60, 941–949. 21067784 10.1016/j.metabol.2010.08.014

[phy270140-bib-0075] Strain, T. , Flaxman, S. , Guthold, R. , Semenova, E. , Cowan, M. , Riley, L. M. , Bull, F. C. , Stevens, G. A. , & Country Data Author G . (2024). National, regional, and global trends in insufficient physical activity among adults from 2000 to 2022: A pooled analysis of 507 population‐based surveys with 5.7 million participants. The Lancet Global Health, 12, e1232–e1243. 38942042 10.1016/S2214-109X(24)00150-5 PMC11254784

[phy270140-bib-0076] Subbotina, E. , Sierra, A. , Zhu, Z. , Gao, Z. , Koganti, S. R. , Reyes, S. , Stepniak, E. , Walsh, S. A. , Acevedo, M. R. , Perez‐Terzic, C. M. , Hodgson‐Zingman, D. M. , & Zingman, L. V. (2015). Musclin is an activity‐stimulated myokine that enhances physical endurance. Proceedings of the National Academy of Sciences of the United States of America, 112, 16042–16047. 26668395 10.1073/pnas.1514250112 PMC4702977

[phy270140-bib-0077] Subramanian, A. , Tamayo, P. , Mootha, V. K. , Mukherjee, S. , Ebert, B. L. , Gillette, M. A. , Paulovich, A. , Pomeroy, S. L. , Golub, T. R. , Lander, E. S. , & Mesirov, J. P. (2005). Gene set enrichment analysis: A knowledge‐based approach for interpreting genome‐wide expression profiles. Proceedings of the National Academy of Sciences of the United States of America, 102, 15545–15550. 16199517 10.1073/pnas.0506580102 PMC1239896

[phy270140-bib-0078] Teich, T. , Dunford, E. C. , Porras, D. P. , Pivovarov, J. A. , Beaudry, J. L. , Hunt, H. , Belanoff, J. K. , & Riddell, M. C. (2016). Glucocorticoid antagonism limits adiposity rebound and glucose intolerance in young male rats following the cessation of daily exercise and caloric restriction. American Journal of Physiology. Endocrinology and Metabolism, 311, E56–E68. 27143556 10.1152/ajpendo.00490.2015 PMC4967147

[phy270140-bib-0015] The Galaxy Community . (2024). The galaxy platform for accessible, reproducible, and collaborative data analyses: 2024 update. Nucleic Acids Research, 52, W83–W94. 38769056 10.1093/nar/gkae410 PMC11223835

[phy270140-bib-0079] Thyfault, J. P. , & Krogh‐Madsen, R. (1985). Metabolic disruptions induced by reduced ambulatory activity in free‐living humans. Journal of Applied Physiology, 111, 1218–1224. 10.1152/japplphysiol.00478.2011 21636564

[phy270140-bib-0080] Trim, W. V. , Walhin, J. P. , Koumanov, F. , Turner, J. E. , Shur, N. F. , Simpson, E. J. , Macdonald, I. A. , Greenhaff, P. L. , & Thompson, D. (2023). The impact of physical inactivity on glucose homeostasis when diet is adjusted to maintain energy balance in healthy, young males. Clinical Nutrition, 42, 532–540. 36857962 10.1016/j.clnu.2023.02.006

[phy270140-bib-0081] Tsai, A. C. , Bach, J. , & Borer, K. T. (1981). Somatic, endocrine, and serum lipid changes during detraining in adult hamsters. The American Journal of Clinical Nutrition, 34, 373–376. 7010986 10.1093/ajcn/34.3.373

[phy270140-bib-0082] Wang, Q. , Cao, D. H. , Jin, C. L. , Lin, C. K. , Ma, H. W. , & Wu, Y. Y. (2011). A method of utrophin up‐regulation through RNAi‐mediated knockdown of the transcription factor EN1. The Journal of International Medical Research, 39, 161–171. 21672318 10.1177/147323001103900117

[phy270140-bib-0083] Wang, Y. , Jin, G. , Guo, Y. , Cao, Y. , Niu, S. , Fan, X. , & Zhang, J. (2021). SMYD2 suppresses p53 activity to promote glucose metabolism in cervical cancer. Experimental Cell Research, 404, 112649. 34015314 10.1016/j.yexcr.2021.112649

[phy270140-bib-0084] Wang, Z. , Zhu, S. , Jia, Y. , Wang, Y. , Kubota, N. , Fujiwara, N. , Gordillo, R. , Lewis, C. , Zhu, M. , Sharma, T. , Li, L. , Zeng, Q. , Lin, Y. H. , Hsieh, M. H. , Gopal, P. , Wang, T. , Hoare, M. , Campbell, P. , Hoshida, Y. , & Zhu, H. (2023). Positive selection of somatically mutated clones identifies adaptive pathways in metabolic liver disease. Cell, 186, 1968. 37040760 10.1016/j.cell.2023.03.014 PMC10321862

[phy270140-bib-0085] Watts, A. J. , & Storey, K. B. (2019). Hibernation impacts lysine methylation dynamics in the 13‐lined ground squirrel, Ictidomys tridecemlineatus. Journal of Experimental Zoology Part A, Ecological and Integrative Physiology, 331, 234–244. 30767414 10.1002/jez.2259

[phy270140-bib-0086] Wickham, H. , Averick, M. , Bryan, J. , Chang, W. , D'Agostino McGowan, L. , François, R. , Grolemund, G. , Hayes, A. , Henry, L. , Hester, J. , Kuhn, M. , Lin Pedersen, T. , Miller, E. , Milton Bache, S. , Müller, K. , Ooms, J. , Robinson, D. , Seidel, D. P. , Spinu, V. , … Yutani, H. (2019). Welcome to the Tidyverse. Journal of Open Source Software, 4, 1686.

[phy270140-bib-0087] Wijndaele, K. , Orrow, G. , Ekelund, U. , Sharp, S. J. , Brage, S. , Griffin, S. J. , & Simmons, R. K. (2014). Increasing objectively measured sedentary time increases clustered cardiometabolic risk: A 6 year analysis of the ProActive study. Diabetologia, 57, 305–312. 24194101 10.1007/s00125-013-3102-y PMC3889989

[phy270140-bib-0088] World Health Organization . (2020). WHO guidelines on physical activity and sedentary behaviour. World Health Organization.

[phy270140-bib-0089] Yue, F. , Cheng, Y. , Breschi, A. , Vierstra, J. , Wu, W. , Ryba, T. , Sandstrom, R. , Ma, Z. , Davis, C. , Pope, B. D. , Shen, Y. , Pervouchine, D. D. , Djebali, S. , Thurman, R. E. , Kaul, R. , Rynes, E. , Kirilusha, A. , Marinov, G. K. , Williams, B. A. , … Mouse, E. C. (2014). A comparative encyclopedia of DNA elements in the mouse genome. Nature, 515, 355–364. 25409824 10.1038/nature13992 PMC4266106

[phy270140-bib-0090] Zhang, C. , Weng, Y. , Shi, F. , & Jin, W. (2016). The Engrailed‐1 gene stimulates Brown Adipogenesis. Stem Cells International, 2016, 7369491. 27148369 10.1155/2016/7369491 PMC4842372

[phy270140-bib-0091] Zhang, M. , Du, S. , Ou, H. , Cui, R. , Jiang, N. , Lin, Y. , Ge, R. , Ma, D. , & Zhang, J. (2021). Ablation of Zfhx4 results in early postnatal lethality by disrupting the respiratory center in mice. Journal of Molecular Cell Biology, 13, 210–224. 33475140 10.1093/jmcb/mjaa081 PMC8260053

[phy270140-bib-0092] Zou, P. , Chen, C. , & Wu, X. (2024). Identification of fatty acid oxidation‐related subtypes by integrated analysis of bulk‐ and single‐cell transcriptome profiling in colorectal cancer. J Gastrointest Oncol, 15, 147–163. 38482228 10.21037/jgo-23-833 PMC10932680

